# Oncogenic Potential of Hepatitis C Virus Proteins

**DOI:** 10.3390/v2092108

**Published:** 2010-09-27

**Authors:** Arup Banerjee, Ratna B. Ray, Ranjit Ray

**Affiliations:** 1 Department of Internal Medicine, Edward A. Doisy Research Center, 1100 S. Grand Blvd., 8th Floor, St. Louis, MO 63104, USA; E-Mail: abanerj1@slu.edu; 2 Department of Pathology, Edward A. Doisy Research Center, 1100 S. Grand Blvd., 2nd Floor, St. Louis, MO 63104, USA; E-Mail: rayrb@slu.edu; 3 Molecular Microbiology & Immunology, Edward A. Doisy Research Center, 1100 S. Grand Blvd., 8th Floor, St. Louis, MO 63104, USA

**Keywords:** Hepatitis C virus, transcriptional regulation, oncogene regulation, microRNA, oxidative stress, apoptosis, fibrosis, metabolic disorders, cytokine modulation, hepatocyte growth regulation hepatocellular carcinoma

## Abstract

Chronic hepatitis C virus (HCV) infection is a major risk factor for liver disease progression, and may lead to cirrhosis and hepatocellular carcinoma (HCC). The HCV genome contains a single-stranded positive sense RNA with a cytoplasmic lifecycle. HCV proteins interact with many host-cell factors and are involved in a wide range of activities, including cell cycle regulation, transcriptional regulation, cell proliferation, apoptosis, lipid metabolism, and cell growth promotion. Increasing experimental evidences suggest that HCV contributes to HCC by modulating pathways that may promote malignant transformation of hepatocytes. At least four of the 10 HCV gene products, namely core, NS3, NS5A and NS5B play roles in several potentially oncogenic pathways. Induction of both endoplasmic reticulum (ER) stress and oxidative stress by HCV proteins may also contribute to hepatocyte growth promotion. The current review identifies important functions of the viral proteins connecting HCV infections and potential for development of HCC. However, most of the putative transforming potentials of the HCV proteins have been defined in artificial cellular systems, and need to be established relevant to infection and disease models. The new insight into the mechanisms for HCV mediated disease progression may offer novel therapeutic targets for one of the most devastating human malignancies in the world today.

## 1. Introduction

Over 200 million people are estimated to be infected with hepatitis C virus (HCV) worldwide, reflecting the unique capacity of this virus to establish long-standing, persistent infection. Within the United States, HCV infection is the leading cause of chronic hepatitis and cirrhosis, and is an increasingly important factor in the etiology of hepatocellular carcinoma (HCC) [1]. Increased incidence of HCC observed over the past several decades is due to an expansion in the number of individuals chronically infected with HCV [2,3]. HCC typically develops often in the setting of cirrhosis, although the underlying mechanisms for cancer progression remain poorly defined.

While genetic alterations are the predominant mechanisms of oncogenesis, viruses have evolved additional methods to affect the same critical pathways in an attempt to promote viral replication. Viruses often encode proteins that modulate normal cellular processes favoring viral replication [4]. Genomic expression profiling studies have identified varying gene expression for HCC associated with HCV infection [5–7]. HCV mediated HCC may reflect distinct molecular mechanisms, including alteration of normal cellular signaling pathways to stimulate host cell growth, and cellular transformation. Indirect mechanisms, including long-standing hepatic inflammation with associated oxidative stress and the potential for DNA damage, are also likely to contribute to the development of HCC [8–10]. There are strong evidences that the HCV proteins (including core, NS3, NS5A and NS5B) potentiate oncogenic transformation. Expression of these HCV proteins, alone or together, promotes growth, when stably expressed in cells or in transgenic mice [11–16]. The consequences of the host immune response to HCV infection, including immune mediated destruction of infected hepatocytes that induces repeated liver regeneration cycles, may as well be involved in disease progression to HCC. In this review, we discuss how multiple interactions of HCV proteins, especially the core protein, with host-cells contribute to the development of liver cancer in chronically infected patients.

## 2. HCV genome organization, protein synthesis and life cycle

**Figure 1.**
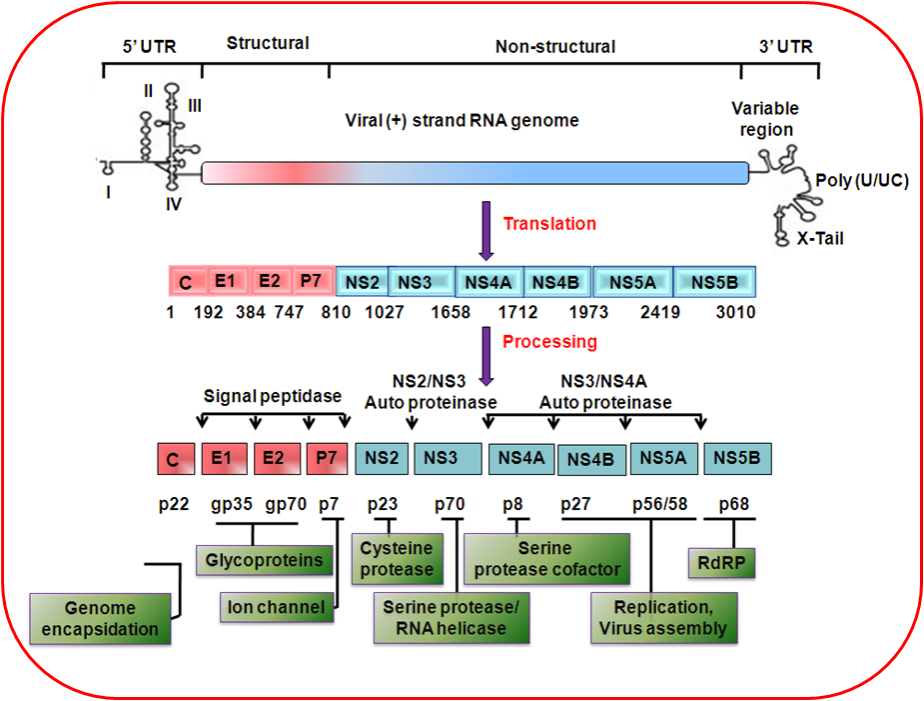
Genomic organization and function of the proteins encoded by HCV.

HCV is classified within the genus *Hepacivirus*, and belongs to the family *Flaviviridae*. The genome of the virus is ∼9.6 kb long and contains a long open reading frame, flanked by untranslated 5′ and 3′ sequences (Figure 1). The untranslated 5′ and 3′ sequences are important for translation and replication of the viral RNA [17,18]. Six genotypes and more than 50 subtypes have been reported based on HCV genomic sequence variations [19,20]. The positive-strand RNA genome of the virus encodes a single large polyprotein that is co- and post-translationally processed by cellular and viral proteases into at least 10 structural and nonstructural (Core, E1, E2/p7, NS2, NS3, NS4A, NS4B, NS5A and NS5B) viral proteins. Proteins derived from the amino-terminal third of the viral polyprotein include the three structural proteins: core and two envelope glycoproteins, E1 and E2.

HCV core is a basic protein with RNA-binding activity that is thought to comprise the nucleocapsid of the virus. Several forms of the core protein of variable molecular weights (17–23 kDa) have been identified [21–24]. Synthesis of proteins encoded from alternative open reading frames from the core genomic region has also been shown [25–28]. However, we did not observe sharing of the major properties of core protein with the translated protein from its alternate open reading frame [29]. HCV core protein has been detected in various subcellular compartments, including cytosol, lipid droplets, endoplasmic reticulum/golgi apparatus, mitochondria, and nuclei. The broad intracellular distribution raises the possibility that HCV core protein may modulate multiple cellular processes [30]. HCV envelope glycoproteins interact with multiple cell surface molecules and LDL-R for orchestration of virus entry into mammalian cells [31,32]. Downstream of these proteins is p7, a small transmembrane protein with ion channel activity. NS2, a non-structural protein, plays a critical role in polyprotein processing and virus assembly. The remaining non-structural proteins (NS3, NS4A, NS4B, NS5A, and NS5B) are required for viral RNA replication [33]. NS3 is a serine protease that is responsible for *cis* or *trans* cleavage at four sites within the HCV polyprotein, thereby generating the amino termini of NS4A, NS4B, NS5A, and NS5B [34]. NS3 also functions as an RNA helicase and NTPase, and is an essential component of the RNA replicase complex [35,36]. NS4A, a small 54-amino-acid protein, forms a stable complex with the amino-terminal third of NS3, protease domain, and is required for complete serine protease activity [37]. NS4B, an integral membrane protein, is mostly localized on the cytoplasmic side of the ER membrane and is implicated in assembly of the replicase complex on lipid rafts [38,39]. NS5A, a phosphoprotein, plays a role in viral resistance to interferon [40,41]. NS5A also plays a role in RNA replication, and virus assembly [42]. NS5B is the RNA-dependent RNA polymerase, and acts as the catalytic core of the macromolecular replicase complex essential for HCV RNA replication [43,44].

Experimental findings using cloned HCV gene expression in mammalian cells, the development of subgenomic or full-length replicon derived from HCV, and the generation of infectious HCV genotypes 1a and 2a in human hepatocyte derived cell lines [45–52] have significantly contributed to the advancement of HCV research. Recently, autophagy has gained importance as it plays an important role in HCV life cycle. We and others have shown that HCV induces autophagy in hepatocytes [53–56]. HCV may induce accumulation of autophagosomes via the induction of ER stress and the unfolded protein response [54,57]. Similar to poliovirus and coxsackieviruses, the induction of autophagosomes may play an important role in HCV replication, as siRNA-knockdown of autophagy related cellular genes, including Atg7, LC3, Atg4B, Atg12 and Beclin-1, altered HCV RNA replication levels [54–56].

## 3. Transcriptional modulation and oncogene regulation by HCV

HCV core protein may directly and indirectly interact with numerous transcription factors, including heterogeneous nuclear ribonucleoprotein K [58], leucine zipper transcription factor (LZIP) [59], 14-3-3 protein [60], RNA helicase CAP-Rf [61], p53 [62], p21 [63,64], and NF-kB [65], and RNA helicase DEAD box DDX3 protein [66,67]. HCV core protein aberrantly sequesters LZIP in the cytoplasm to inactivate its function, and potentiates cellular transformation [59]. Development of HCC might be associated with activation of the Ras/Raf/MAP kinase pathway [68]. The 14-3-3 protein family is known to associate with components of several signal transduction pathways, including the Raf-1 kinase cascade [60]. HCV core protein activates Raf-1 kinase through interaction with 14-3-3 protein family. Thus, HCV core protein may play an important role in regulating hepatocyte growth, senescence, and differentiation through its interaction with 14-3-3 protein. Constitutive expression of HCV core protein results in a high basal activity of MAP kinase kinase, as determined by immunodetection of hyperphosphorylated ERK-1 and ERK-2 [69]. HCV core protein also represses p21 promoter activity [63]. Interaction between HCV core protein and DEAD Box protein DDX3 may be involved in HCV replication [70] although recent studies suggest that the requirement of DDX3 for replication is unrelated to its interaction with the viral core protein [71]. HCV core can also modulate the expression of the cyclin-dependent inhibitor p21, which is a major target of p53 and regulates the activities of cyclin/cyclin-dependent kinase complexes involved in cell-cycle control and tumor formation [63,72,73]. HCV core protein suppresses NF-κB activity in TNF-α, PMA, OA, and H_2_O_2_ treated cells; while upregulates AP1 [69]. Whether activation of AP-1 and suppression of NF-κB by the HCV core are linked to activation of the MAPK pathway is not clear. HCV core protein also selectively reduces phosphorylated STAT1 accumulation in the nucleus in a proteasome-dependent manner, and impairs IFN-α induced signal transduction via expression of suppressor of cytokine signaling (SOCS)-3 [74–76].

Wnt/β-catenin pathway plays a major role in HCC carcinogenesis [77]. The transcriptional upregulation of both wnt-1 and its downstream target WISP-2 by HCV core protein suggested possible activation of the wnt-1 signaling pathway in the promotion of cell growth. NS5A expression in the context of HCV polyprotein inhibits the Akt substrate Forkhead transcription factor and stimulates the phosphorylation of glycogen synthase kinase-3β, leading to stabilization of cellular β-catenin and stimulation of β-catenin-responsive transcription [78,79]. NS5A protein thus may directly be involved in Wnt/β-catenin-mediated liver pathogenesis

We and others have reported transcriptional regulation of cellular genes, such as p53, c-myc and hTERT by HCV core protein [8,30,62,63,69,80–84]. Expression of wild type p53 significantly diminishes STAT3 phosphorylation, STAT3 DNA binding activity, and inhibits STAT3-dependent transcriptional activity [85]. HCV NS5A activates STAT3 in Huh-7 cells [86] and in transgenic mouse liver [87]. STAT3 activation may not only provide a growth advantage [80,88], but also confer resistance to conventional therapies that rely on the apoptotic machinery to eliminate tumor cells.

## 4. Hepatocyte growth regulation by HCV proteins

HCV proteins promote cell proliferation by interfering with cellular proteins involved in different phases of the cell cycle. Normal progression through the cell cycle is regulated by sequential activation of cyclin and cyclin-dependent kinase (CDK) complexes. Active cyclin-CDK complexes in G1 phosphorylate the retinoblastoma family of proteins (pRb, p130, and p107) allowing the release of E2F transcription factors and upregulation of cellular genes to positively reinforce progression through this phase of the cell cycle [89]. Importantly, these checkpoints require active p53 and Rb pathways [90,91].

During the G1/S transition, p53 activates transcription of p21 which, in turn, binds to and inhibits CDK2, causing cell cycle arrest while the cell attempts to repair the DNA damage. Anti-growth signals such as checkpoint activation can limit the replication of oncogenic viruses, particularly if the checkpoint is activated in response to viral infection. We and others have shown that HCV NS5A physically associates with p53 and downregulates the cell cycle regulatory gene p21 [92–94]. Another recent study found that the NS5A protein downregulates the expression of the mitotic spindle protein ASPM through the PKR-p38 signaling pathway and induces aberrant mitoses, chromosome instability and HCC [95].

HCV proteins can bind to p53 [96], p73 [97], and pRb [98,99], but the functional consequences of these interactions have not fully been elucidated. HCV core interacts with p73, causes nuclear translocation of core protein and prevents p73-α-dependent cell growth arrest in a p53-dependent manner [97]. Conditional expression of the core protein results in a decreased abundance of Rb in immortalized rat embryo fibroblasts, leading to enhanced E2F transcription-factor activity [98].

NS5B has been shown to form a cytoplasmic complex with Rb in infected cells [99]. NS5B dependent downregulation of Rb leads both to activation of E2F-dependent transcription and to increased cellular proliferation. Another cell-cycle checkpoint, the mitotic spindle checkpoint (MSC), is also a target for HCV proteins. Significantly, the integrity of Rb appears to be particularly important in the normally quiescent hepatocyte, as liver-specific loss of Rb has been shown to promote ectopic cell-cycle entry and aberrant ploidy [100], which likely contributes to neoplastic transformation. The interaction of the HCV polymerase NS5B with Rb results in the degradation of Rb and activates the *MAD2* promoter [15]. Thus, infection with HCV may lead to a loss of host-cell genomic stability due to deregulation of Rb pathway.

Cross-talk between cellular protein and HCV core protein may be a major risk factor for potentiating HCC. HCV core protein expression alone in a transgenic mouse model was sufficient to induce tumor formation in liver [16]. HCV core can induce spontaneous, persistent, age dependent and heterogeneous activation of PPARα, which may contribute to HCC [101–103]. We also observed that introduction of HCV core protein stimulates primary human hepatocytes to escape from replicative senescence and promotes an immortalized phenotype [104]. Cells retaining an immortalized phenotype display a weak level of core protein expression and exhibit continuous growth. Reactivation of telomerase was observed in the immortalized hepatocytes. HCV core protein introduction resulted in an increase in expression of IL-6, gp130, leptin receptor, and STAT3 [105]. Upregulation of these genes in turn may regulate c-myc and cyclin D1, downstream of the STAT3 signaling pathway, promoting cellular transformation. Repression of the core gene expression in immortalized hepatocytes by a construct of the antisense orientation of the core gene under the control of an inducible metallothionine promoter resulted in apoptosis and characteristic changes in p53, c-myc, and hTERT expression [84]. However, immortalized hepatocytes passaged for a longer time did not display apoptosis from expression of antisense core, likely due to anchorage dependent growth on soft agar, thus proceeding to a transformed phenotype.

A direct role of NS3 was reported in the neoplastic transformation of hepatocytes *in vivo* and *in vitro* [106,107]. Transformation and tumorigenicity occurs upon transfection with HCV NS3 DNA in the non-tumorigenic mouse fibroblast cell line NIH 3T3 into nude mice. HCV NS3 C-terminal-deleted protein also showed transforming and oncogenic potential [108]. Stable expression of the NS3 protein in human hepatocytes induced transformed characters with reduced population doubling time, anchorage-independent growth and tumor development with increase expression of phospho-p44/42 and phospho-p38 proteins. The NS3 protein also forms complexes with p53 [109], and inhibits p21 promoter activity. The NS3 domain of protease and helicase/NTP-ase activity was responsible for the inhibition of p21.

## 5. Role of HCV proteins in cytokine modulation

Various components of the host immune system are involved in the pathogenesis and outcome of HCV infection. There has been an increasing recognition of the roles played by the cell mediated response, especially the cytokine systems; in the immunopathogenesis of chronic hepatitis C. Disease progression due to persistent HCV infection is usually associated with an imbalance between pro-inflammatory and anti-inflammatory cytokines. The development and resolution of an inflammatory process are regulated by a complex interplay between cytokines that have pro- and anti-inflammatory effects [110]. Conflicting data exists concerning the cytokine profile associated with the development of HCC in chronic HCV infection. Some investigators report that the development of HCC in the cirrhotic liver is associated with a predominant Th-2 cytokine profile with increased IL-10 expression [110]. HCV also subverts cellular immunity by inducing IL-10, which in turn inhibits the activation of dendritic cells (DC) and development of Th-1 cells [111,112]. Similarly, there are reports of increased Th-1 cytokine in the setting of HCC [113].

A recent study has highlighted the relationship between the activation of genes involved in the IL-6 signaling pathway and the development of HCC [114]. An increase in the β-2 microglobulin serum level as well as IL-6 level was observed among HCV infected HCC patients. Weakening of the immune system, due to IL-6, may be responsible for a more severe progression of HCC and the hyperexpression of β-2 microglobulin [115]. We have recently shown that HCV core protein attenuates IL-6 stimulated acute-phase response, and may contribute to impaired innate immunity for viral persistence [116]. TNF-α plays a diverse role in HCV infection. Activation of TNF-α has a pivotal role in the inflammatory process of chronic hepatitis C, and TNF-α levels correlate with the degree of inflammation [117,118].

HCV core also upregulates the expression of TGF-β [119,120], and NS5A modulates TGF-β signaling through interaction with TGF-β receptor I [121]. As HCV-infected livers progress from chronic hepatitis to cirrhosis and/or HCC, hepatocytic pSmad3L/PAI-1 increases with fibrotic stage and necroinflammatory grade, and pSmad3C/p21 decreases [122]. Therefore, it is possible that chronic inflammation associated with HCV infection shifts hepatocytic TGF-β signaling from tumor suppression to fibrogenesis, accelerating liver fibrosis and increasing the risk of HCC. Another study showed that different thresholds of Smad3 activation control TGF-β responses in hepatocytes and that liver cancer-derived HCV core protein, by decreasing Smad3 activation, switches TGF-β growth inhibitory effects to tumor-promoting responses [123]. A recent study found that HCV core triggers the production of both TGF-β2 and VEGF proteins through multiple pathways, including PKC, RB/E2F1, ASK1-JNK/p38 and ERK [124]. HCV core protein also behaves as a positive regulator in androgen receptor signaling and enhances the expression of VEGF in hepatocytes [125].

## 6. Role of HCV in oxidative stress and apoptosis

Oxidative stress induced by HCV infection plays a role in the pathogenesis of liver disease. HCV core protein induces oxidative DNA damage, while it inhibits apoptosis accompanied by enhanced ROS production [126,127], indicating two independent functional aspects. Inducible nitric oxide synthase (iNOS) is upregulated in HCV core introduced hepatocytes [105]. iNOS induces the production of total nitric oxide (NO) from L-arginine in inflamed tissues. NO plays an important role in many physiological and pathological conditions, serving as an intercellular and intracellular messenger and antimicrobial agent [128]. NO induces DNA cleavage, and enhances the chance of mutation. This sequence of events may contribute to HCV mediated pathogenesis and oncogenesis [129]. Oxidative stress leads indirectly from DNA damage to p53 induction, which can lead to activation of BAX and apoptosis [130,131].

Apoptosis is a key element in a host organism’s defense against viral infections, inhibiting viral spread and persistence. Alterations in cell survival contribute to the pathogenesis of a number of human diseases, including viral oncogenesis [132]. During HCV infection, hepatocyte apoptosis could be induced by immune attack on infected cells or directly by viral infection. Hepatocyte damage plays a role in the recruitment and activation of stellate cells and macrophages and the subsequent development of fibrosis [133,134]. HCV infected patients have higher levels of immune related death ligands; TRAIL, TNF-α, FAS, and FASL [135–137]. The expression of HCV proteins may inhibit Fas mediated apoptosis and death in mice by repressing the release of Cyt-C from mitochondria, thereby suppressing caspases-9 and -3/7 activation [138]. At least two HCV viral proteins (Core and NS5A) play an important role in modulation of apoptosis [139,140]. Our observations suggest that HCV NS5A protein impairs TNF mediated apoptosis, but not by Fas antibody, in a transgenic mouse model [141]. HCV nonstructural proteins are the key mediators of sensitization to TRAIL. Sensitization to TRAIL was shown to be caspase-9 dependent and mediated in part via the mitochondrial pathway [142], and may contribute to the elimination of virus infected hepatocytes. Earlier study by our group suggested that HCV core mediates a novel mechanism of apoptosis, in which hepatocytes death correlates with an increase in Apaf-1 [143]. The subsequent activation of caspase-9, leading to the initiation of the intrinsic cell death pathway, occurs in the absence of cytochrome c translocation to the cytosol. HCV core protein suppresses apoptosis mediated by TNF-α [144]. A sustained expression of c-FLIP, an endogenous caspase-8 inhibitor, inhibits TNF-α induced apoptotic pathway in HCV core expressing hepatocytes [145]. Apoptotic activity of common chemotherapeutic drugs (5-fluorouracil, doxorubicin or cisplatin) or chemotherapeutic cytokine are highly dependent on the status of p53 [146,147]. HCV core protein mediated modulation of p53 may protect cells from chemotherapeutic drug induced apoptosis, allowing cancer cells to proliferate or survive inappropriately. Cytokine or drug induced apoptosis is modulated by HCV core protein in different cells [63,69,144,148]. We have recently identified an association between HCV core and cellular HAX-1 proteins, which may promote 5-FU mediated p53-dependent caspase-7 activation and hepatocyte growth inhibition [149]. p53 is a critical component for apoptosis. HCV NS5A has also been shown to inhibit p53 induced apoptosis [92,94,150,151]. NS5A interacts with and partially sequesters p53 and hTAF (II), a component of TFIID and an essential coactivator of p53, and suppresses p53-mediated transcriptional activation and apoptosis [152]. NS5A also forms complexes with the TBP and p53 and inhibits the binding of both p53 and TBP to their DNA consensus binding sequences *in vitro*. Further, this may inhibit p53-TBP and p53-excision repair cross complementing factor 3 protein–protein complex formations [94]. NS5A interacts also with Bax as a Bcl-2 homolog and prevents apoptosis in a p53-independent manner [153].

Expression of either HCV genome or individual HCV structural protein (core or E1) induces endoplasmic reticulum (ER) stress [154,155] and the unfolded protein response (UPR), which can lead to apoptosis. Recently, HCV infection in chimeric *SCID/Alb-uPA* mice correlated with increased levels of the ER chaperone GPR78/BiP, a key regulator of the unfolded protein response. In addition, levels of pro-apoptotic BAX were increased, while anti-apoptotic NF-κB and BCL-xL were decreased in HCV infected cells [156]. Therefore, ER stress induced by HCV combined with lower NF-κB and BCL-xL levels may sensitize hepatocytes to apoptosis.

## 7. HCV associated metabolic disorders and liver disease progression

The metabolic syndrome is a constellation of problems that includes insulin resistance, obesity, hypertension, and hyperlipidemia. Increasingly, components of the metabolic syndrome are being linked to various forms of cancer with respect to both increased risk of disease and worsened outcome. Experimental studies indicate that insulin resistance occurring in HCV core-transgenic mice is due at least partly to an increase in TNF-α secretion [157]. TNF-α has also systemic effects that result in insulin resistance and type 2 diabetes (T2D). Marked increases in both sTNFR1 and sTNFR2 were demonstrated in HCV-diabetic patients [158]. Possible explanations for the unique association between insulin resistance and HCV infection may be related to differences in the clinical course of liver inflammation and fibrosis, or in the mode of TNF-receptor activation or cleavage. Thus, in the correlation between liver disease and insulin resistance, a link among chronic HCV infection, TNF-α, and T2D possibly exists [159,160].

We have observed that HCV core protein alone or together with other viral proteins upregulates serine phosphorylation of insulin receptor substrate-1 and impair the downstream Akt/protein kinase B signaling pathway for insulin resistance [161]. Insulin resistance is paradoxically associated with a reduced ability of insulin signaling to inhibit glucose production, whereas insulin-stimulated lipogenesis is enhanced in the liver and two transcription factors, FoxO1 and FoxA2 play an important role in this process. A recent study on 165 consecutive patients with newly diagnosed HCC suggested that insulin resistance is associated with HCC in chronic hepatitis C infection [162]. We have shown that HCV can differentially modulate activation of forkhead transcription factors and insulin induced metabolic gene expression [163].

Insulin resistance and subsequent hyperinsulinemia are highly associated with fatty liver disease and is an important risk factor for the progression of fibrosis in chronic hepatitis C [160,164,165]. From the metabolic aspect, hepatitis C resembles non-alcoholic steatohepatitis (NASH) in numerous features, such as the presence of steatosis, serum dyslipidemia, and oxidative stress in the liver [166]. In contrast, there are noticeable differences between hepatitis C and NASH, in that HCV modulates cellular gene expression and intracellular signal transduction, while such details have not been noted for NASH. A recent report suggests that HCV may actively contribute to the fibrogenic process via the paracrine effect of IL-8 secreted by infected hepatocytes. [167].

HCV core protein expression leads to the development of progressive hepatic steatosis (fatty change) and HCC in transgenic mice [168]. Persistent activation of PPARα has also been suggested for the pathogenesis of hepatic steatosis and hepatocellular carcinoma in HCV core expressing transgenic mice [102]. Hepatic steatosis occurs at a high rate (40–86%) in chronic HCV patients, and a close relationship between steatosis and intrahepatic core protein expression has been noted [169]. Insulin resistance is a prominent mechanism linking steatosis and fibrogenesis although this link is complex and poorly understood.

Hepatic stellate cells (HSCs) are one of the sinusoid constituent cells that play multiple roles in liver pathophysiology and, in particular, in liver fibrosis [170]. Liver fibrosis is one of the major complications associated with HCV infection, but the mechanism underlying the molecular basis of HCV-related fibrosis is unclear. Progressive liver fibrosis may eventually lead to cirrhosis and HCC. Insulin resistance is a significant risk factor for hepatic fibrosis in patients with chronic HCV, either directly or by favoring hepatic steatosis. HCV infection generates oxidative stress, TNF-α, and IL-6 production in the liver. Oxidative stress and these cytokines are well known profibrogenic mediators [171]. HCV may induce fibrosis directly either by stimulating secretion of profibrogenic cytokines by hepatocytes, by interacting with sinusoidal endothelium, or by directly provoking fibrogenesis by HSCs.

## 8. Induction of miRNAs by HCV

miRNAs affect gene silencing via both translational inhibition and mRNA degradation [172]. The expression of host cell miRNAs can be modulated by HCV. Several studies have shown that the expression of miRNAs is altered in human HCC, implicating them in hepatocarcinogenesis. [173]. Abnormally expressed miRNAs may work as functional actors in HCC initiation and progression. miRNAs that are unique to certain virus-related HCC have been identified. By comparing HCV-HCC tissues and adjacent non HCC tissues, 29 differentially expressed miRNAs were identified [174]. Nineteen of these miRNAs are differentially expressed between HBV-HCC and HCV-HCC [175]. Since most of these miRNAs are HCC-associated, these results suggest dual roles of miRNAs in viral replication and HCC development. Among several mRNAs modulated in HCV infected liver tissues, the function of miR122 was extensively studied. MiR-122 enhances HCV RNA translation [176–180]. Studies of miRNA expression in liver tissues of HCV-infected patients showed increased expression of several miRNAs, including miR-122 in HCC tissues when compared with normal adjacent tissues, suggesting that the underlying HCV infection can modulate the expression of miRNAs in cancer [174]. Contrary to this finding of miR-122 upregulation in HCV associated HCC; other investigators have reported a down regulation in hepatoma cell lines with etiologies other than HCV infection [174,181]. Because miR122 closely interacts with the HCV genome and miR-122 expression pattern in HCV associated HCC is directly opposed to non-HCV infected HCC, we speculate that HCV infected transformed hepatocytes are able to circumvent tumorigenic repression of miR-122. HCV dependent modulation of miRNAs expression including miR-122 was also studied in HCV expressing hepatoma cell lines [179,182]. Interestingly, cell culture study reveals that miR-122 is downregulated (∼3 fold) during acute HCV infection [182].

Recently, Peng *et al.* [183] carried out a computational study of HCV associated miRNAs-mRNA regulatory modules in human livers. They found differential profile of cellular miRNAs that target the genes involved in chemokine, B cell receptor, PTEN, IL-6, ERK/MAPK and JAK/STAT signaling pathways, suggesting a critical role of miRNAs in the replication, propagation, and latency of virus in the host cell. Upregulation of miR-155 was correlated with the growth promotion of HCC cells [184], and HCV replication associates with an increase in expression of cholesterol biosynthesis genes that are regulated by miR-122 [185]. Together, these findings suggest that miRNAs have the potential to become novel drug targets in virally induced infectious or malignant diseases.

## 9. Cooperative interactions of HCV and other agents in promoting liver disease

HCV increases the risk for HCC by promoting the development of liver fibrosis and cirrhosis. The question remains whether HCV causes HCC directly or promotes as a cooperative oncogene for end stage liver disease progression. HCC arising from a noncirrhotic liver vary according to geographic location (0% to 68.4%), and represents an uncommon and poorly defined subgroup of HCC [186]. Several studies suggested that patients infected with HCV genotype 1b have more rapid progression of associated liver dysfunction and a 2–6 fold increased risk for HCC [187]. Viral proteins, including HCV core, play important role in liver disease associated with infection [188–190]. The variables affecting the range of pathology induced by HCV and the widely differing rates of disease progression are poorly understood and are likely to be multi-factorial, including aspects of host genetics, immune responses, diet, and alcohol consumption. Viral factors such as viral load, genotype, and variation within individual viral genes may as well affect the range of pathology. Several studies suggest that HCC might be a hormone-responsive neoplasm, and the role of sex hormone receptors in primary liver tumors have been implicated [191]. Androgen receptor (AR) expression is detected with more intense expression in HCC than in non-tumoral liver tissue. Our recent study demonstrated that HCV core protein alone or in context with other HCV proteins enhances AR-mediated transcriptional activity and further augments in the presence of androgen [125]. Subsequent study suggested that HCV core protein acts as a positive regulator in AR signaling, providing further insight into oncogenic potential in the development of HCC in HCV infected individuals.

Dual infection with HCV and HBV in cirrhotic patients has been linked to an increased risk of HCC. A meta-analysis of case-control studies found a synergism between the two viruses with regard to carcinogenesis, the risk being more additive than multiplicative [192,193]. In cohort studies among Italian or Chinese patients with cirrhosis, those with HCV/HBV coinfection had a two- to six-fold higher risk of developing HCC compared with those with single infection [194]. HCC occurs at a younger age and after a shorter period of HCV infection in subjects coinfected with human immunodeficiency virus (HIV) compared with patients with HCV related HCC but without HIV infection [195]. Since newer therapies are decreasing mortality from HIV infection, it is anticipated that an increase in the incidence of HCC will appear in the future among HCV/HIV coinfected persons. Case-control studies have shown that there is more than additive interaction between alcohol and HCV infection in the development of HCC [196–198]. Together, these reports suggest that cooperative interactions of other agents with HCV have prolonged effect on HCV induced liver pathogenesis.

## 10. Summary

**Figure 2.**
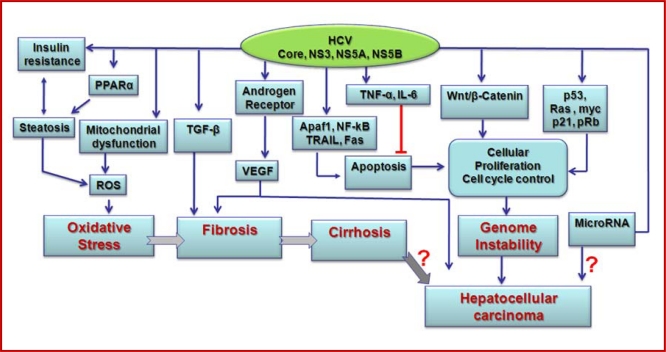
Schematic view of the molecular mechanisms for HCV mediated end stage liver disease progression.

Chronic HCV infection is a major risk factor for the development of end stage liver disease, including HCC. Immune mediated liver damage may occur from HCV infected hepatocyte death and rapid turnover of hepatocytes with altered genetic changes for development of HCC. However, hepatocyte death does not appear to occur at a high rate as the liver transaminase upregulation is modest and intermittent during HCV chronicity. We have highlighted some of the major effects of HCV proteins promoting cell growth with the potential for oncogenesis (Figure 2). While the transcriptional and cellular effects from HCV are well studied, there are still gaps in our understanding of how HCV influences oncogenesis. Many intriguing functions related to HCV core protein, which may significantly contribute to disease progression have been reported.

Alterations in cell cycle proteins and their regulation are clearly involved in cancer progression and cellular transformation pathways. Activities of the HCV proteins are thought to contribute to the development of HCV associated promotion of hepatocyte growth, which may develop into HCC. Further understanding of the cellular factors targeted by HCV proteins and their effects on viral replication and cellular components of the liver could provide new insight and provide a better understanding of the development of liver cancer in chronically HCV infected patients. HCV appears to program hepatocyte cell machinery for viral replication and growth promotion towards the development of HCC. Most of the putative transforming potentials of the HCV proteins have been defined in artificial cellular systems, which may not be applicable to HCV infection *in vivo*, and still need to be established relevant to infection and disease models. Unfortunately, we are yet to develop a suitable small animal disease model from HCV infection. Thus, the true biologic relevance of these observations remains still to be established in a relevant infection model scenario.

HCC arising from a noncirrhotic liver, although uncommon, suggests that this disease process may follow a distinct pathway, independent of cirrhosis. Genetic and environmental factors and other cooperative agents may be involved with HCC. HCV proteins interact with a number of host factors and signaling pathways, and thus contribute to the progression from chronic hepatitis C to liver cirrhosis and HCC. However, it is difficult to demonstrate specific roles of HCV proteins *in vivo*, and in the microenvironment due to the lack of a suitable animal model. Role of miRNAs in viral life cycle is an emerging field, and future studies will elude their specific role in HCV mediated pathogenesis. As HCV mediated liver disease progression is slow and often takes more than a decade, there is longtime for treatment opportunity. Thus, we hope understanding the mechanism for liver disease progression from chronic HCV infection would offer opportunity for optimum treatment and intervention strategies.

## References

[1] El-Serag HB (2004). Hepatocellular carcinoma: recent trends in the United States. Gastroenterology.

[2] Kiyosawa K, Umemura T, Ichijo T, Matsumoto A, Yoshizawa K, Gad A, Tanaka E (2004). Hepatocellular carcinoma: recent trends in Japan. Gastroenterology.

[3] Umemura T, Ichijo T, Yoshizawa K, Tanaka E, Kiyosawa K (2009). Epidemiology of hepatocellular carcinoma in Japan. J Gastroenterol.

[4] Gearhart TL, Bouchard MJ (2010). The hepatitis B virus X protein modulates hepatocyte proliferation pathways to stimulate viral replication. J Virol.

[5] Iizuka N, Oka M, Yamada-Okabe H, Mori N, Tamesa T, Okada T, Takemoto N, Tangoku A, Hamada K, Nakayama H, Miyamoto T, Uchimura S, Hamamoto Y (2002). Comparison of gene expression profiles between hepatitis B virus- and hepatitis C virus-infected hepatocellular carcinoma by oligonucleotide microarray data on the basis of a supervised learning method. Cancer Res.

[6] Yoon SY, Kim JM, Oh JH, Jeon YJ, Lee DS, Kim JH, Choi JY, Ahn BM, Kim S, Yoo HS, Kim YS, Kim NS (2006). Gene expression profiling of human HBV- and/or HCV-associated hepatocellular carcinoma cells using expressed sequence tags. Int J Oncol.

[7] De Giorgi V, Monaco A, Worchech A, Tornesello M, Izzo F, Buonaguro L, Marincola FM, Wang E, Buonaguro FM (2009). Gene profiling, biomarkers and pathways characterizing HCV-related hepatocellular carcinoma. J Transl Med.

[8] Nakamoto Y, Guidotti LG, Kuhlen CV, Fowler P, Chisari FV (1998). Immune pathogenesis of hepatocellular carcinoma. J Exp Med.

[9] Okuda M, Li K, Beard MR, Showalter LA, Scholle F, Lemon SM, Weinman SA (2002). Mitochondrial injury, oxidative stress, and antioxidant gene expression are induced by hepatitis C virus core protein. Gastroenterology.

[10] Bartsch H, Nair J (2004). Oxidative stress and lipid peroxidation-derived DNA-lesions in inflammation driven carcinogenesis. Cancer Detect Prev.

[11] Ray RB, Lagging LM, Meyer K, Ray R (1996). Hepatitis C virus core protein cooperates with ras and transforms primary rat embryo fibroblasts to tumorigenic phenotype. J Virol.

[12] Gale M, Kwieciszewski B, Dossett M, Nakao H, Katze MG (1999). Antiapoptotic and oncogenic potentials of hepatitis C virus are linked to interferon resistance by viral repression of the PKR protein kinase. J Virol.

[13] Park JS, Yang JM, Min MK (2000). Hepatitis C virus nonstructural protein NS4B transforms NIH3T3 cells in cooperation with the Ha-ras oncogene. Biochem Biophys Res Commun.

[14] Lerat H, Honda M, Beard MR, Loesch K, Sun J, Yang Y, Okuda M, Gosert R, Xiao SY, Weinman SA, Lemon SM (2002). Steatosis and liver cancer in transgenic mice expressing the structural and nonstructural proteins of hepatitis C virus. Gastroenterology.

[15] Munakata T, Nakamura M, Liang Y, Li K, Lemon SM (2005). Down-regulation of the retinoblastoma tumor suppressor by the hepatitis C virus NS5B RNA-dependent RNA polymerase. Proc Natl Acad Sci U S A.

[16] Moriya K, Fujie H, Shintani Y, Yotsuyanagi H, Tsutsumi T, Ishibashi K, Matsuura Y, Kimura S, Miyamura T, Koike K (1998). The core protein of hepatitis C virus induces hepatocellular carcinoma in transgenic mice. Nat Med.

[17] Friebe P, Lohmann V, Krieger N, Bartenschlager R (2001). Sequences in the 5’nontranslated region of hepatitis C virus required for RNA replication. J Virol.

[18] Yi M, Lemon SM (2003). 3’ nontranslated RNA signals required for replication of hepatitis C virus RNA. J Virol.

[19] Simmonds P (1996). Virology of hepatitis C virus. Clin Ther.

[20] Simmonds P (2001). The origin and evolution of hepatitis viruses in humans. J Gen Virol.

[21] Harada S, Watanabe Y, Takeuchi K, Suzuki T, Katayama T, Takebe Y, Saito I, Miyamura T (1991). Expression of processed core protein of hepatitis C virus in mammalian cells. J Virol.

[22] Ray RB, Lagging LM, Meyer K, Steele R, Ray R (1995). Transcriptional regulation of cellular and viral promoters by the hepatitis C virus core protein. Virus Res.

[23] Liu Q, Tackney C, Bhat RA, Prince AM, Zhang P (1997). Regulated processing of hepatitis C virus core protein is linked to subcellular localization. J Virol.

[24] Yasui K, Wakita T, Tsukiyama-Kohara K, Funahashi SI, Ichikawa M, Kajita T, Moradpour D, Wands JR, Kohara M (1998). The native form and maturation process of hepatitis C virus core protein. J Virol.

[25] Xu Z, Choi J, Yen TS, Lu W, Strohecker A, Govindarajan S, Chien D, Selby MJ, Ou J (2001). Synthesis of a novel hepatitis C virus protein by ribosomal frameshift. EMBO J.

[26] Walewski JL, Keller TR, Stump DD, Branch AD (2001). Evidence for a new hepatitis C virus antigen encoded in an overlapping reading frame. RNA.

[27] Varaklioti A, Vassilaki N, Georgopoulou U, Mavromara P (2002). Alternate translation occurs within the core coding region of the hepatitis C viral genome. J Biol Chem.

[28] Vassilaki N, Mavromara P (2003). Two alternative translation mechanisms are responsible for the expression of the HCV ARFP/F/core+1 coding open reading frame. J Biol Chem.

[29] Basu A, Steele R, Ray R, Ray RB (2004). Functional properties of a 16 kDa protein translated from an alternative open reading frame of the core-encoding genomic region of hepatitis C virus. J Gen Virol.

[30] Ray RB, Ray R (2001). Hepatitis C virus core protein: intriguing properties and functional relevance. FEMS Microbiol Lett.

[31] Meyer K, Basu A, Ray R (2000). Functional features of hepatitis C virus glycoproteins for pseudotype virus entry into mammalian cells. Virology.

[32] Meyer K, Beyene A, Bowlin TL, Basu A, Ray R (2004). Coexpression of hepatitis C virus E1 and E2 chimeric envelope glycoproteins displays separable ligand sensitivity and increases pseudotype infectious titer. J Virol.

[33] Lohmann V, Körner F, Koch J, Herian U, Theilmann L, Bartenschlager R (1999). Replication of subgenomic hepatitis C virus RNAs in a hepatoma cell line. Science.

[34] Tomei L, Failla C, Santolini E, De Francesco R, La Monica N (1993). NS3 is a serine protease required for processing of hepatitis C virus polyprotein. J Virol.

[35] Suzich JA, Tamura JK, Palmer-Hill F, Warrener P, Grakoui A, Rice CM, Feinstone SM, Collett MS (1993). Hepatitis C virus NS3 protein polynucleotide-stimulated nucleoside triphosphatase and comparison with the related pestivirus and flavivirus enzymes. J Virol.

[36] Gosert R, Egger D, Lohmann V, Bartenschlager R, Blum HE, Bienz K, Moradpour D (2003). Identification of the hepatitis C virus RNA replication complex in Huh-7 cells harboring subgenomic replicons. J Virol.

[37] Bhattacherjee V, Prescott LE, Pike I, Rodgers B, Bell H, El-Zayadi AR, Kew MC, Conradie J, Lin CK, Marsden H (1995). Use of NS-4 peptides to identify type-specific antibody to hepatitis C virus genotypes 1, 2, 3, 4, 5 and 6. J Gen Virol.

[38] Hugle T, Fehrmann F, Bieck E, Kohara M, Krausslich HG, Rice CM, Blum HE, Moradpour D (2001). The hepatitis C virus nonstructural protein 4B is an integral endoplasmic reticulum membrane protein. Virology.

[39] Gao L, Aizaki H, He JW, Lai MM (2004). Interactions between viral nonstructural proteins and host protein hVAP-33 mediate the formation of hepatitis C virus RNA replication complex on lipid raft. J Virol.

[40] Enomoto N, Sakuma I, Asahina Y, Kurosaki M, Murakami T, Yamamoto C, Ogura Y, Izumi N, Marumo F, Sato C (1996). Mutations in the nonstructural protein 5A gene and response to interferon in patients with chronic hepatitis C virus 1b infection. N. Engl J Med.

[41] Gale MJ, Korth MJ, Tang NM, Tan SL, Hopkins DA, Dever TE, Polyak SJ, Gretch DR, Katze MG (1997). Evidence that hepatitis C virus resistance to interferon is mediated through repression of the PKR protein kinase by the nonstructural 5A protein. Virology.

[42] Appel N, Zayas M, Miller S, Krijnse-Locker J, Schaller T, Friebe P, Kallis S, Engel U, Bartenschlager R (2008). Essential role of domain III of nonstructural protein 5A for hepatitis C virus infectious particle assembly. PLoS Pathog.

[43] Behrens SE, Tomei L, De Francesco R (1996). Identification and properties of the RNA-dependent RNA polymerase of hepatitis C virus. EMBO J.

[44] Lohmann V, Roos A, Körner F, Koch JO, Bartenschlager R (1998). Biochemical and kinetic analyses of NS5B RNA-dependent RNA polymerase of the hepatitis C virus. Virology.

[45] Pietschmann T, Lohmann V, Kaul A, Krieger N, Rinck G, Rutter G, Strand D, Bartenschlager R (2002). Persistent and transient replication of full-length hepatitis C virus genomes in cell culture. J Virol.

[46] Zhong J, Gastaminza P, Cheng G, Kapadia S, Kato T, Burton DR, Wieland SF, Uprichard SL, Wakita T, Chisari FV (2005). Robust hepatitis C virus infection *in vitro*. Proc Natl Acad Sci U S A.

[47] Wakita T, Pietschmann T, Kato T, Date T, Miyamoto M, Zhao Z, Murthy K, Habermann A, Kräusslich HG, Mizokami M, Bartenschlager R, Liang TJ (2005). Production of infectious hepatitis C virus in tissue culture from a cloned viral genome. Nat Med.

[48] Lindenbach BD, Evans MJ, Syder AJ, Wölk B, Tellinghuisen TL, Liu CC, Maruyama T, Hynes RO, Burton DR, McKeating JA, Rice CM (2005). Complete replication of hepatitis C virus in cell culture. Science.

[49] Cai Z, Zhang C, Chang KS, Jiang J, Ahn BC, Wakita T, Liang TJ, Luo G (2005). Robust production of infectious hepatitis C virus (HCV) from stably HCV cDNA-transfected human hepatoma cells. J Virol.

[50] Heller T, Saito S, Auerbach J, Williams T, Moreen TR, Jazwinski A, Cruz B, Jeurkar N, Sapp R, Luo G, Liang TJ (2005). An *in vitro* model of hepatitis C virion production. Proc Natl Acad Sci U S A.

[51] Kanda T, Basu A, Steele R, Wakita T, Ryerse JS, Ray R, Ray RB (2006). Generation of infectious hepatitis C virus in immortalized human hepatocytes. J Virol.

[52] Yi M, Villanueva RA, Thomas DL, Wakita T, Lemon SM (2006). Production of infectious genotype 1a hepatitis C virus (Hutchinson strain) in cultured human hepatoma cells. Proc Natl Acad Sci U S A.

[53] Ait-Goughoulte M, Kanda T, Meyer K, Ryerse JS, Ray RB, Ray R (2008). Hepatitis C virus genotype 1a growth and induction of autophagy. J Virol.

[54] Sir D, Liang C, Chen WL, Jung JU, Ou JH (2008). Perturbation of autophagic pathway by hepatitis C virus. Autophagy.

[55] Dreux M, Chisari FV (2009). Autophagy proteins promote hepatitis C virus replication. Autophagy.

[56] Tanida I, Fukasawa M, Ueno T, Kominami E, Wakita T, Hanada K (2009). Knockdown of autophagy-related gene decreases the production of infectious hepatitis C virus particles. Autophagy.

[57] Sir D, Chen WL, Choi J, Wakita T, Yen TS, Ou JH (2008). Induction of incomplete autophagic response by hepatitis C virus via the unfolded protein response. Hepatology.

[58] Hsieh TY, Matsumoto M, Chou HC, Schneider R, Hwang SB, Lee AS, Lai MM (1998). Hepatitis C virus core protein interacts with heterogeneous nuclear ribonucleoprotein K. J Biol Chem.

[59] Jin DY, Wang HL, Zhou Y, Chun AC, Kibler KV, Hou YD, Kung H, Jeang KT (2000). Hepatitis C virus core protein-induced loss of LZIP function correlates with cellular transformation. EMBO J.

[60] Aoki H, Hayashi J, Moriyama M, Arakawa Y, Hino O (2000). Hepatitis C virus core protein interacts with 14-3-3 protein and activates the kinase Raf-1. J Virol.

[61] You LR, Chen CM, Lee YH (1999). Hepatitis C virus core protein enhances NF-kappaB signal pathway triggering by lymphotoxin-beta receptor ligand and tumor necrosis factor alpha. J Virol.

[62] Ray RB, Steele R, Meyer K, Ray R (1997). Transcriptional repression of p53 promoter by hepatitis C virus core protein. J Biol Chem.

[63] Ray RB, Steele R, Meyer K, Ray R (1998). Hepatitis C virus core protein represses p21WAF1/Cip1/Sid1 promoter activity. Gene.

[64] Wang F, Yoshida I, Takamatsu M, Ishido S, Fujita T, Oka K, Hotta H (2000). Complex formation between hepatitis C virus core protein and p21Waf1/Cip1/Sdi1. Biochem Biophys Res Commun.

[65] Liao QJ, Ye LB, Timani KA, She YL, Yang XJ, Ye L, Wu ZH (2005). Hepatitis C virus non-structural 5A protein can enhance full-length core protein-induced nuclear factor-kappaB activation. World J Gastroenterol.

[66] Mamiya N, Worman HJ (1999). Hepatitis C virus core protein binds to a DEAD box RNA helicase. J Biol Chem.

[67] Owsianka AM, Patel AH (1999). Hepatitis C virus core protein interacts with a human DEAD box protein DDX3. Virology.

[68] Ito Y, Sasaki Y, Horimoto M, Wada S, Tanaka Y, Kasahara A, Ueki T, Hirano T, Yamamoto H, Fujimoto J, Okamoto E, Hayashi N, Hori M (1998). Activation of mitogen-activated protein kinases/extracellular signal-regulated kinases in human hepatocellular carcinoma. Hepatology.

[69] Shrivastava A, Manna SK, Ray R, Aggarwal BB (1998). Ectopic expression of hepatitis C virus core protein differentially regulates nuclear transcription factors. J Virol.

[70] Ariumi Y, Kuroki M, Abe K, Dansako H, Ikeda M, Wakita T, Kato N (2007). DDX3 DEAD-box RNA helicase is required for hepatitis C virus RNA replication. J Virol.

[71] Angus AG, Dalrymple D, Boulant S, McGivern DR, Clayton RF, Scott MJ, Adair R, Graham S, Owsianka AM, Targett-Adams P, Li K, Wakita T, McLauchlan J, Lemon SM, Patel AH (2010). Requirement of cellular DDX3 for hepatitis C virus replication is unrelated to its interaction with the viral core protein. J Gen Virol.

[72] Wagayama H, Shiraki K, Sugimoto K, Ito T, Fujikawa K, Yamanaka T, Takase K, Nakano T (2002). High expression of p21WAF1/CIP1 is correlated with human hepatocellular carcinoma in patients with hepatitis C virus-associated chronic liver diseases. Hum Pathol.

[73] Kwun HJ, Jang KL (2003). Dual effects of hepatitis C virus Core protein on the transcription of cyclin-dependent kinase inhibitor p21 gene. J Viral Hepat.

[74] Bode JG, Ludwig S, Ehrhardt C, Albrecht U, Erhardt A, Schaper F, Heinrich PC, Häussinger D (2003). IFN-alpha antagonistic activity of HCV core protein involves induction of suppressor of cytokine signaling-3. FASEB J.

[75] Vlotides G, Sörensen AS, Kopp F, Zitzmann K, Cengic N, Brand S, Zachoval R, Auernhammer CJ (2004). SOCS-1 and SOCS-3 inhibit IFN-alpha-induced expression of the antiviral proteins 2,5-OAS and MxA. Biochem Biophys Res Commun.

[76] Lin W, Choe WH, Hiasa Y, Kamegaya Y, Blackard JT, Schmidt EV, Chung RT (2005). Hepatitis C virus expression suppresses interferon signaling by degrading STAT1. Gastroenterology.

[77] Fukutomi T, Zhou Y, Kawai S, Eguchi H, Wands JR, Li J (2005). Hepatitis C virus core protein stimulates hepatocyte growth: correlation with upregulation of wnt-1 expression. Hepatology.

[78] Street A, Macdonald A, McCormick C, Harris M (2005). Hepatitis C virus NS5A-mediated activation of phosphoinositide 3-kinase results in stabilization of cellular beta-catenin and stimulation of beta-catenin-responsive transcription. J Virol.

[79] Park CY, Choi SH, Kang SM, Kang JI, Ahn BY, Kim H, Jung G, Choi KY, Hwang SB (2009). Nonstructural 5A protein activates beta-catenin signaling cascades: implication of hepatitis C virus-induced liver pathogenesis. J Hepatol.

[80] Yoshida T, Hanada T, Tokuhisa T, Kosai K, Sata M, Kohara M, Yoshimura A (2002). Activation of STAT3 by the hepatitis C virus core protein leads to cellular transformation. J Exp Med.

[81] Ray RB, Ray R, Tabor E (2002). Perspective in medical virology. Virus and liver cancer.

[82] Bergqvist A, Rice CM (2001). Transcriptional activation of the interleukin-2 promoter by hepatitis C virus core protein. J Virol.

[83] Lai MM, Ware CF (2000). Hepatitis C virus core protein: possible roles in viral pathogenesis. Curr Top Microbiol Immunol.

[84] Basu A, Meyer K, Ray RB, Ray R (2002). Hepatitis C virus core protein is necessary for the maintenance of immortalized human hepatocytes. Virology.

[85] Lin J, Tang H, Jin X, Jia G, Hsieh JT (2002). p53 regulates Stat3 phosphorylation and DNA binding activity in human prostate cancer cells expressing constitutively active Stat3. Oncogene.

[86] Waris G, Turkson J, Hassanein T, Siddiqui A (2005). Hepatitis C virus (HCV) constitutively activates STAT-3 via oxidative stress: role of STAT-3 in HCV replication. J Virol.

[87] Sarcar B, Ghosh AK, Steele R, Ray R, Ray RB (2004). Hepatitis C virus NS5A mediated STAT3 activation requires co-operation of Jak1 kinase. Virology.

[88] Bromberg J, Darnell JE (2000). The role of STATs in transcriptional control and their impact on cellular function. Oncogene.

[89] Massagué J (2004). G1 cell-cycle control and cancer. Nature.

[90] Agarwal ML, Agarwal A, Taylor WR, Stark GR (1995). p53 controls both the G2/M and the G1 cell cycle checkpoints and mediates reversible growth arrest in human fibroblasts. Proc Natl Acad Sci U S A.

[91] Harrington EA, Bruce JL, Harlow E, Dyson N (1998). pRB plays an essential role in cell cycle arrest induced by DNA damage. Proc Natl Acad Sci U S A.

[92] Majumder M, Ghosh AK, Steele R, Ray R, Ray RB (2001). Hepatitis C virus NS5A physically associates with p53 and regulates p21/waf1 gene expression in a p53-dependent manner. J Virol.

[93] Lan KH, Sheu ML, Hwang SJ, Yen SH, Chen SY, Wu JC, Wang YJ, Kato N, Omata M, Chang FY, Lee SD (2002). HCV NS5A interacts with p53 and inhibits p53-mediated apoptosis. Oncogene.

[94] Qadri I, Iwahashi M, Simon F (2002). Hepatitis C virus NS5A protein binds TBP and p53, inhibiting their DNA binding and p53 interactions with TBP and ERCC3. Biochim Biophys Acta.

[95] Wu SC, Chang SC, Wu HY, Liao PJ, Chang MF (2008). Hepatitis C virus NS5A protein downregulates the expression of spindle gene Aspm through PKR-p38 signaling pathway. J Biol Chem.

[96] Lu W, Lo SY, Chen M, Wu K, Fung YK, Ou JH (1999). Activation of p53 tumor suppressor by hepatitis C virus core protein. Virology.

[97] Alisi A, Giambartolomei S, Cupelli F, Merlo P, Fontemaggi G, Spaziani A, Balsano C (2003). Physical and functional interaction between HCV core protein and the different p73 isoforms. Oncogene.

[98] Cho J, Baek W, Yang S, Chang J, Sung YC, Suh M (2001). HCV core protein modulates Rb pathway through pRb down-regulation and E2F-1 up-regulation. Biochim Biophys Acta.

[99] Munakata T, Liang Y, Kim S, McGivern DR, Huibregtse J, Nomoto A, Lemon SM (2007). Hepatitis C virus induces E6AP-dependent degradation of the retinoblastoma protein. PLoS Pathog.

[100] Machida K, Liu JC, McNamara G, Levine A, Duan L, Lai MM (2009). Hepatitis C virus causes uncoupling of mitotic checkpoint and chromosomal polyploidy through the Rb pathway. J Virol.

[101] Mayhew CN, Bosco EE, Fox SR, Okaya T, Tarapore P, Schwemberger SJ, Babcock GF, Lentsch AB, Fukasawa K, Knudsen ES (2005). Liver-specific pRB loss results in ectopic cell cycle entry and aberrant ploidy. Cancer Res.

[102] Miyamoto H, Moriishi K, Moriya K, Murata S, Tanaka K, Suzuki T, Miyamura T, Koike K, Matsuura Y (2007). Involvement of the PA28gamma-dependent pathway in insulin resistance induced by hepatitis C virus core protein. J Virol.

[103] Tanaka N, Moriya K, Kiyosawa K, Koike K, Gonzalez FJ, Aoyama T (2008). PPARalpha activation is essential for HCV core protein-induced hepatic steatosis and hepatocellular carcinoma in mice. J Clin Invest.

[104] Koike K (2009). Steatosis, liver injury, and hepatocarcinogenesis in hepatitis C viral infection. J Gastroenterol.

[105] Ray RB, Meyer K, Ray R (2000). Hepatitis C virus core protein promotes immortalization of primary human hepatocytes. Virology.

[106] Basu A, Meyer K, Lai KK, Saito K, Di Bisceglie AM, Grosso LE, Ray RB, Ray R (2006). Microarray analyses and molecular profiling of Stat3 signaling pathway induced by hepatitis C virus core protein in human hepatocytes. Virology.

[107] Sakamuro D, Furukawa T, Takegami T (1995). Hepatitis C virus nonstructural protein NS3 transforms NIH 3T3 cells. J Virol.

[108] Zemel R, Gerechet S, Greif H, Bachmatove L, Birk Y, Golan-Goldhirsh A, Kunin M, Berdichevsky Y, Benhar I, Tur-Kaspa R (2001). Cell transformation induced by hepatitis C virus NS3 serine protease. J Viral Hepat.

[109] He QQ, Cheng RX, Sun Y, Feng DY, Chen ZC, Zheng H (2003). Hepatocyte transformation and tumor development induced by hepatitis C virus NS3 c-terminal deleted protein. World J Gastroenterol.

[110] Ishido S, Fujita T, Hotta H (1998). Complex formation of NS5B with NS3 and NS4A proteins of hepatitis C virus. Biochem Biophys Res Commun.

[111] Zekri AR, Ashour MS, Alam El-Din, HM, Khaled HM, Abu-Shady M (2005). Cytokines as markers for disease progression in HCV associated liver diseases. World J Gastroenterol.

[112] Brady MT, MacDonald AJ, Rowan AG, Mills KH (2003). Hepatitis C virus non-structural protein 4 suppresses Th1 responses by stimulating IL-10 production from monocytes. Eur J Immunol.

[113] Saito K, Ait-Goughoulte M, Truscott SM, Meyer K, Blazevic A, Abate G, Ray RB, Hoft DF, Ray R (2008). Hepatitis C virus inhibits cell surface expression of HLA-DR, prevents dendritic cell maturation, and induces interleukin-10 production. J Virol.

[114] Zekri AR, Bahnassy AA, Abdel-Wahab SA, Khafagy MM, Loutfy SA, Radwan H, Shaarawy SM (2009). Expression of pro- and anti-inflammatory cytokines in relation to apoptotic genes in Egyptian liver disease patients associated with HCV-genotype-4. J Gastroenterol Hepatol.

[115] Tang Y, Kitisin K, Jogunoori W, Li C, Deng CX, Mueller SC, Ressom HW, Rashid A, He AR, Mendelson JS, Jessup JM, Shetty K, Zasloff M, Mishra B, Reddy EP, Johnson L, Mishra L (2008). Progenitor/stem cells give rise to liver cancer due to aberrant TGF-beta and IL-6 signaling. Proc Natl Acad Sci U S A.

[116] Malaguarnera M, Di Fazio I, Ferlito L, Pistone G, Laurino A, Vinci E, Mazzoleni G (2000). Increase of serum beta2-microglobulin in patients affected by HCV correlated hepatocellular carcinoma. Eur J Gastroenterol Hepatol.

[117] Ait-Goughoulte M, Banerjee A, Meyer K, Mazumdar B, Saito K, Ray RB, Ray R (2010). Hepatitis C virus core protein interacts with fibrinogen-beta and attenuates cytokine stimulated acute-phase response. Hepatology.

[118] Tilg H, Wilmer A, Vogel W, Herold M, Nolchen B, Judmaier G, Huber C (1992). Serum levels of cytokines in chronic liver diseases. Gastroenterology.

[119] Torre D, Zeroli M, Giola G, Ferrario G, Fiori P, Bonetta G, Tambini R (1994). Serum levels of interleukin-1 alpha, interleukin-1 beta, interleukin-6, and tumor necrosis factor in patients with acute viral hepatitis. Clin Infect Dis.

[120] Taniguchi H, Kato N, Otsuka M, Goto T, Yoshida H, Shiratori Y, Omata M (2004). Hepatitis C virus core protein upregulates transforming growth factor-beta 1 transcription. J Med Virol.

[121] Shin JY, Hur W, Wang JS, Jang JW, Kim CW, Bae SH, Jang SK, Yang SH, Sung YC, Kwon OJ, Yoon SK (2005). HCV core protein promotes liver fibrogenesis via up-regulation of CTGF with TGF-beta1. Exp Mol Med.

[122] Choi SH, Hwang SB (2006). Modulation of the transforming growth factor-beta signal transduction pathway by hepatitis C virus nonstructural 5A protein. J Biol Chem.

[123] Matsuzaki K, Murata M, Yoshida K, Sekimoto G, Uemura Y, Sakaida N, Kaibori M, Kamiyama Y, Nishizawa M, Fujisawa J, Okazaki K, Seki T (2007). Chronic inflammation associated with hepatitis C virus infection perturbs hepatic transforming growth factor beta signaling, promoting cirrhosis and hepatocellular carcinoma. Hepatology.

[124] Battaglia S, Benzoubir N, Nobilet S, Charneau P, Samuel D, Zignego AL, Atfi A, Bréchot C, Bourgeade MF (2009). Liver cancer-derived hepatitis C virus core proteins shift TGF-beta responses from tumor suppression to epithelial-mesenchymal transition. PLoS One.

[125] Hassan M, Selimovic D, Ghozlan H, Abdel-kader O (2009). Hepatitis C virus core protein triggers hepatic angiogenesis by a mechanism including multiple pathways. Hepatology.

[126] Kanda T, Steele R, Ray R, Ray RB (2008). Hepatitis C virus core protein augments androgen receptor-mediated signaling. J Virol.

[127] Tardif KD, Waris G, Siddiqui A (2005). Hepatitis C virus, ER stress, and oxidative stress. Trends Microbiol.

[128] Li Y, Boehning DF, Qian T, Popov VL, Weinman SA (2007). Hepatitis C virus core protein increases mitochondrial ROS production by stimulation of Ca2+ uniporter activity. FASEB J.

[129] Machida K, Cheng KT, Sung VM, Lee KJ, Levine AM, Lai MM (2004). Hepatitis C virus infection activates the immunologic (type II) isoform of nitric oxide synthase and thereby enhances DNA damage and mutations of cellular genes. J Virol.

[130] Castello G, Scala S, Palmieri G, Curley SA, Izzo F (2010). HCV-related hepatocellular carcinoma: From chronic inflammation to cancer. Clin Immunol.

[131] Chipuk JE, Kuwana T, Bouchier-Hayes L, Droin NM, Newmeyer DD, Schuler M, Green DR (2004). Direct activation of Bax by p53 mediates mitochondrial membrane permeabilization and apoptosis. Science.

[132] Fan J, Ren H, Jia N, Fei E, Zhou T, Jiang P, Wu M, Wang G (2008). DJ-1 decreases Bax expression through repressing p53 transcriptional activity. J Biol Chem.

[133] Thompson CB (1995). Apoptosis in the pathogenesis and treatment of disease. Science.

[134] Canbay A, Higuchi H, Bronk SF, Taniai M, Sebo TJ, Gores GJ (2002). Fas enhances fibrogenesis in the bile duct ligated mouse: a link between apoptosis and fibrosis. Gastroenterology.

[135] Bataller R, Brenner DA (2005). Liver fibrosis. J Clin Invest.

[136] Pianko S, Patella S, Ostapowicz G, Desmond P, Sievert W (2001). Fas-mediated hepatocyte apoptosis is increased by hepatitis C virus infection and alcohol consumption, and may be associated with hepatic fibrosis: mechanisms of liver cell injury in chronic hepatitis C virus infection. J Viral Hepat.

[137] Mundt B, Wirth T, Zender L, Waltemathe M, Trautwein C, Manns MP, Kühnel F, Kubicka S (2005). Tumour necrosis factor related apoptosis inducing ligand (TRAIL) induces hepatic steatosis in viral hepatitis and after alcohol intake. Gut.

[138] Riordan SM, Skinner NA, Kurtovic J, Locarnini S, McIver CJ, Williams R, Visvanathan K (2006). Tolllike receptor expression in chronic hepatitis C: correlation with proinflammatory cytokine levels and liver injury. Inflamm Res.

[139] Machida K, Tsukiyama-Kohara K, Seike E, Toné S, Shibasaki F, Shimizu M, Takahashi H, Hayashi Y, Funata N, Taya C, Yonekawa H, Kohara M (2001). Inhibition of cytochrome c release in Fas-mediated signaling pathway in transgenic mice induced to express hepatitis C viral proteins. J Biol Chem.

[140] Hara Y, Hino K, Okuda M, Furutani T, Hidaka I, Yamaguchi Y, Korenaga M, Li K, Weinman SA, Lemon SM, Okita K (2006). Hepatitis C virus core protein inhibits deoxycholic acid-mediated apoptosis despite generating mitochondrial reactive oxygen species. J Gastroenterol.

[141] Chou AH, Tsai HF, Wu YY, Hu CY, Hwang LH, Hsu PI, Hsu PN (2005). Hepatitis C virus core protein modulates TRAIL-mediated apoptosis by enhancing Bid cleavage and activation of mitochondria apoptosis signaling pathway. J Immunol.

[142] Majumder M, Ghosh AK, Steele R, Zhou XY, Phillips NJ, Ray R, Ray RB (2002). Hepatitis C virus NS5A protein impairs TNF-mediated hepatic apoptosis, but not by an anti-FAS antibody, in transgenic mice. Virology.

[143] Lan L, Gorke S, Rau SJ, Zeisel MB, Hildt E, Himmelsbach K, Carvajal-Yepes M, Huber R, Wakita T, Schmitt-Graeff A, Royer C, Blum HE, Fischer R, Baumert TF (2008). Hepatitis C virus infection sensitizes human hepatocytes to TRAIL-induced apoptosis in a caspase 9-dependent manner. J Immunol.

[144] Meyer K, Basu A, Saito K, Ray RB, Ray R (2005). Inhibition of hepatitis C virus core protein expression in immortalized human hepatocytes induces cytochrome c-independent increase in Apaf-1 and caspase-9 activation for cell death. Virology.

[145] Ray RB, Meyer K, Steele R, Shrivastava A, Aggarwal BB, Ray R (1998). Inhibition of tumor necrosis factor (TNF-alpha)-mediated apoptosis by hepatitis C virus core protein. J Biol Chem.

[146] Saito K, Meyer K, Warner R, Basu A, Ray RB, Ray R (2006). Hepatitis C virus core protein inhibits tumor necrosis factor alpha-mediated apoptosis by a protective effect involving cellular FLICE inhibitory protein. J Virol.

[147] Nagane M, Huang HJ, Cavenee WK (2001). The potential of TRAIL for cancer chemotherapy. Apoptosis.

[148] Meurette O, Huc L, Rebillard A, Le Moigne G, Lagadic-Gossmann D, Dimanche-Boitrel MT (2005). TRAIL (TNF-related apoptosis-inducing ligand) induces necrosis-like cell death in tumor cells at acidic extracellular pH. Ann NY Acad Sci.

[149] Ray RB, Meyer K, Ray R (1996). Suppression of apoptotic cell death by hepatitis C virus core protein. Virology.

[150] Banerjee A, Saito K, Meyer K, Banerjee S, Ait-Goughoulte M, Ray RB, Ray R (2009). Hepatitis C virus core protein and cellular protein HAX-1 promote 5-fluorouracil-mediated hepatocyte growth inhibition. J Virol.

[151] Gong GZ, Jiang YF, He Y, Lai LY, Zhu YH, Su XS (2004). HCV NS5A abrogates p53 protein function by interfering with p53-DNA binding. World J Gastroenterol.

[152] Otsuka M, Kato N, Lan K, Yoshida H, Kato J, Goto T, Shiratori Y, Omata M (2000). Hepatitis C virus core protein enhances p53 function through augmentation of DNA binding affinity and transcriptional ability. J Biol Chem.

[153] Lan KH, Sheu ML, Hwang SJ, Yen SH, Chen SY, Wu JC, Wang YJ, Kato N, Omata M, Chang FY, Lee SD (2002). HCV NS5A interacts with p53 and inhibits p53-mediated apoptosis. Oncogene.

[154] Chung YL, Sheu ML, Yen SH (2003). Hepatitis C virus NS5A as a potential viral Bcl-2 homologue interacts with Bax and inhibits apoptosis in hepatocellular carcinoma. Int J Cancer.

[155] Benali-Furet NL, Chami M, Houel L, De Giorgi F, Vernejoul F, Lagorce D, Buscail L, Bartenschlager R, Ichas F, Rizzuto R, Paterlini-Bréchot P (2005). Hepatitis C virus core triggers apoptosis in liver cells by inducing ER stress and ER calcium depletion. Oncogene.

[156] Chan SW, Egan P (2005). A Hepatitis C virus envelope proteins regulate CHOP via induction of the unfolded protein response. FASEB J.

[157] Joyce MA, Walters KA, Lamb SE, Yeh MM, Zhu LF, Kneteman N, Doyle JS, Katze MG, Tyrrell DL (2009). HCV induces oxidative and ER stress, and sensitizes infected cells to apoptosis in SCID/Alb-uPA mice. PLoS Pathog.

[158] Shintani Y, Fujie H, Miyoshi H, Tsutsumi T, Tsukamoto K, Kimura S, Moriya K, Koike K (2004). Hepatitis C virus infection and diabetes: direct involvement of the virus in the development of insulin resistance. Gastroenterology.

[159] Knobler H, Zhornicky T, Sandler A, Haran N, Ashur Y, Schattner A (2003). Tumor necrosis factor-alpha-induced insulin resistance may mediate the hepatitis C virus-diabetes association. Am J Gastroenterol.

[160] Sheikh MY, Choi J, Qadri I, Friedman JE, Sanyal AJ (2008). Hepatitis C virus infection: molecular pathways to metabolic syndrome. Hepatology.

[161] Moucari R, Asselah T, Cazals-Hatem D, Voitot H, Boyer N, Ripault MP, Sobesky R, Martinot-Peignoux M, Maylin S, Nicolas-Chanoine MH, Paradis V, Vidaud M, Valla D, Bedossa P, Marcellin P (2008). Insulin resistance in chronic hepatitis C: association with genotypes 1 and 4, serum HCV RNA level, and liver fibrosis. Gastroenterology.

[162] Banerjee S, Saito K, Ait-Goughoulte M, Meyer K, Ray RB, Ray R (2008). Hepatitis C virus core protein upregulates serine phosphorylation of insulin receptor substrate-1 and impairs the downstream akt/protein kinase B signaling pathway for insulin resistance. J Virol.

[163] Hung CH, Wang JH, Hu TH, Chen CH, Chang KC, Yen YH, Kuo YH, Tsai MC, Lu SN, Lee CM (2010). Insulin resistance is associated with hepatocellular carcinoma in chronic hepatitis C infection. World J Gastroenterol.

[164] Banerjee A, Meyer K, Mazumdar B, Ray RB, Ray R (2010). Hepatitis C virus differentially modulates activation of forkhead transcription factors and insulin induced metabolic gene expression. J Virol.

[165] Ortiz V, Berenguer M, Rayón JM, Carrasco D, Berenguer J (2002). Contribution of obesity to hepatitis C-related fibrosis progression. Am J Gastroenterol.

[166] Bugianesi E, Manzini P, D’Antico S, Vanni E, Longo F, Leone N, Massarenti P, Piga A, Marchesini G, Rizzetto M (2004). Relative contribution of iron burden, HFE mutations, and insulin resistance to fibrosis in nonalcoholic fatty liver. Hepatology.

[167] Koike K, Moriya K (2005). Metabolic aspects of hepatitis C viral infection: steatohepatitis resembling but distinct from NASH. J Gastroenterol.

[168] Clément S, Pascarella S, Conzelmann S, Gonelle-Gispert C, Guilloux K, Negro F (2010). The hepatitis C virus core protein indirectly induces alpha-smooth muscle actin expression in hepatic stellate cells via interleukin-8. J Hepatol.

[169] Moriya K, Fujie H, Shintani Y, Yotsuyanagi H, Tsutsumi T, Ishibashi K, Matsuura Y, Kimura S, Miyamura T, Koike K (1998). The core protein of hepatitis C virus induces hepatocellular carcinoma in transgenic mice. Nat Med.

[170] Fujie H, Yotsuyanagi H, Moriya K, Shintani Y, Tsutsumi T, Takayama T, Makuuchi M, Matsuura Y, Miyamura T, Kimura S, Koike K (1999). Steatosis and intrahepatic hepatitis C virus in chronic hepatitis. J Med Virol.

[171] Friedman SL (2008). Hepatic fibrosis -- overview. Toxicology.

[172] Nieto N (2006). Oxidative-stress and IL-6 mediate the fibrogenic effects of [corrected] Kupffer cells on stellate cells. Hepatology.

[173] Bartel DP (2009). MicroRNAs: target recognition and regulatory functions. Cell.

[174] Junfang Ji, Wang XW (2009). New kids on the block Diagnostic and prognostic microRNAs in hepatocellular carcinoma. Cancer Biology & Therapy.

[175] Varnholt H, Drebber U, Schulze F, Wedemeyer I, Schirmacher P, Dienes HP, Odenthal M (2008). MicroRNA gene expression profile of hepatitis C virus-associated hepatocellular carcinoma. Hepatology.

[176] Ura S, Honda M, Yamashita T, Ueda T, Takatori H, Nishino R, Sunakozaka H, Sakai Y, Horimoto K, Kaneko S (2009). Differential microRNA expression between hepatitis B and hepatitis C leading disease progression to hepatocellular carcinoma. Hepatology.

[177] Jopling CL, Yi M, Lancaster AM, Lemon SM, Sarnow P (2005). Modulation of hepatitis C virus RNA abundance by a liver-specific MicroRNA. Science.

[178] Kutay H, Bai S, Datta J, Motiwala T, Pogribny I, Frankel W, Jacob ST, Ghoshal K (2006). Downregulation of miR-122 in the rodent and human hepatocellular carcinomas. J Cell Biochem.

[179] Jopling CL, Norman K, Sarnow P (2006). Positive and negative modulation of viral and cellular mRNAs by liver-specific microRNA miR-122. Cold Spring Harb Symp Quant Biol.

[180] Randall G, Panis M, Cooper JD, Tellinghuisen TL, Sukhodolets KE, Pfeffer S, Landthaler M, Landgraf P, Kan S, Lindenbach BD, Chien M, Weir DB, Russo JJ, Ju J, Brownstein MJ, Sheridan R, Sander C, Zavolan M, Tuschl T, Rice CM (2007). Cellular cofactors affecting hepatitis C virus infection and replication. Proc Natl Acad Sci U S A.

[181] Lin CJ, Gong HY, Tseng HC, Wang WL, Wu JL (2008). miR-122 targets an anti-apoptotic gene, Bcl-w, in human hepatocellular carcinoma cell lines. Biochem Biophys Res Commun.

[182] Chang J, Nicolas E, Marks D, Sander C, Lerro A, Buendia MA, Xu C, Mason WS, Moloshok T, Bort R, Zaret KS, Taylor JM (2004). miR-122, a mammalian liver-specific microRNA, is processed from hcr mRNA and may downregulate the high affinity cationic amino acid transporter CAT-1. RNA Biol.

[183] Liu X, Wang T, Wakita T, Yang W (2010). Systematic identification of microRNA and messenger RNA profiles in hepatitis C virus-infected human hepatoma cells. Virology.

[184] Peng X, Li Y, Walters KA, Rosenzweig ER, Lederer SL, Aicher LD, Proll S, Katze MG (2009). Computational identification of hepatitis C virus associated microRNA-mRNA regulatory modules in human livers. BMC Genomics.

[185] Wang B, Majumder S, Nuovo G, Kutay H, Volinia S, Patel T, Schmittgen TD, Croce C, Ghoshal K, Jacob ST (2009). Role of microRNA-155 at early stages of hepatocarcinogenesis induced by choline-deficient and amino acid-defined diet in C57BL/6 mice. Hepatology.

[186] Shan Y, Zheng J, Lambrecht RW, Bonkovsky HL (2007). Reciprocal effects of micro-RNA-122 on expression of heme oxygenase-1 and hepatitis C virus genes in human hepatocytes. Gastroenterology.

[187] Madhoun MF, Fazili J, Bright BC, Bader T, Roberts DN, Bronze MS (2010). Hepatitis C prevalence in patients with hepatocellular carcinoma without cirrhosis. Am J Med Sci.

[188] Benvegnù L, Gios M, Boccato S, Alberti A (2004). Natural history of compensated viral cirrhosis: a prospective study on the incidence and hierarchy of major complications. Gut.

[189] Koike K (2007). Hepatitis C virus contributes to hepatocarcinogenesis by modulating metabolic and intracellular signaling pathways. J Gastroenterol Hepatol.

[190] Jin DY (2007). Molecular pathogenesis of hepatitis C virus-associated hepatocellular carcinoma. Front Biosci.

[191] Blonski W, Reddy KR (2008). Hepatitis C virus infection and hepatocellular carcinoma. Clin Liver Dis.

[192] Kalra M, Mayes J, Assefa S, Kaul AK, Kaul R (2008). Role of sex steroid receptors in pathobiology of hepatocellular carcinoma. World J Gastroenterol.

[193] Chiba T, Matsuzaki Y, Abei M, Shoda J, Aikawa T, Tanaka N, Osuga T (1996). Multivariate analysis of risk factors for hepatocellular carcinoma in patients with hepatitis C virus-related liver cirrhosis. J Gastroenterol.

[194] Donato F, Boffetta P, Puoti M (1998). A meta-analysis of epidemiological studies on the combined effect of hepatitis B and C virus infections in causing hepatocellular carcinoma. Int J Cancer.

[195] Fattovich G, Stroffolini T, Zagni I, Donato F (2004). Hepatocellular carcinoma in cirrhosis: incidence and risk factors. Gastroenterology.

[196] Garcìa-Samaniego J, Rodrìquez M, Berenguer J, Rodrìguez-Rosado R, Carbò J, Asensi V, Soriano V (2001). Hepatocellular carcinoma in HIV-infected patients with chronic hepatitis C. Am J Gastroenterol.

[197] El-Serag H, Richardson PA, Everhart JE (2001). The role of diabetes in hepatocellular carcinoma: a case-control study among United States Veterans. Am J Gastroenterol.

[198] Donato F, Tagger A, Gelatti U, Parrinello G, Boffetta P, Albertini A, Decarli A, Travisi P, Ribero ML, Martelli C, Porru S, Nardi G (2002). Alcohol and hepatocellular carcinoma: the effect of lifetime intake and hepatitis virus infections in men and women. Am J Epidemiol.

[199] Hassan MM, Hwang LY, Hatten CJ, Swaim M, Li D, Abbruzzese JL, Beasley P, Patt YZ (2002). Risk factors for hepatocellular carcinoma: synergism of alcohol with viral hepatitis and diabetes mellitus. Hepatology.

